# From Single Cells to Tissues: Interactions between the Matrix and Human Breast Cells in Real Time

**DOI:** 10.1371/journal.pone.0093325

**Published:** 2014-04-01

**Authors:** Clifford Barnes, Lucia Speroni, Kyle P. Quinn, Mael Montevil, Kurt Saetzler, Gbemisola Bode-Animashaun, George McKerr, Irene Georgakoudi, C. Stephen Downes, Carlos Sonnenschein, C. Vyvyan Howard, Ana M. Soto

**Affiliations:** 1 School of Biomedical Sciences, University of Ulster, Coleraine, County Londonderry, United Kingdom; 2 Department of Integrative Physiology and Pathobiology, Tufts University School of Medicine, Boston, Massachusetts, United States of America; 3 Department of Biomedical Engineering, Tufts University, Boston, Massachusetts, United States of America; Université de Technologie de Compiègne, France

## Abstract

**Background:**

Mammary gland morphogenesis involves ductal elongation, branching, and budding. All of these processes are mediated by stroma - epithelium interactions. Biomechanical factors, such as matrix stiffness, have been established as important factors in these interactions. For example, epithelial cells fail to form normal acinar structures *in vitro* in 3D gels that exceed the stiffness of a normal mammary gland. Additionally, heterogeneity in the spatial distribution of acini and ducts within individual collagen gels suggests that local organization of the matrix may guide morphogenesis. Here, we quantified the effects of both bulk material stiffness and local collagen fiber arrangement on epithelial morphogenesis.

**Results:**

The formation of ducts and acini from single cells and the reorganization of the collagen fiber network were quantified using time-lapse confocal microscopy. MCF10A cells organized the surrounding collagen fibers during the first twelve hours after seeding. Collagen fiber density and alignment relative to the epithelial surface significantly increased within the first twelve hours and were a major influence in the shaping of the mammary epithelium. The addition of Matrigel to the collagen fiber network impaired cell-mediated reorganization of the matrix and increased the probability of spheroidal acini rather than branching ducts. The mechanical anisotropy created by regions of highly aligned collagen fibers facilitated elongation and branching, which was significantly correlated with fiber organization. In contrast, changes in bulk stiffness were not a strong predictor of this epithelial morphology.

**Conclusions:**

Localized regions of collagen fiber alignment are required for ductal elongation and branching suggesting the importance of local mechanical anisotropy in mammary epithelial morphogenesis. Similar principles may govern the morphology of branching and budding in other tissues and organs.

## Introduction

At the end of the 19^th^ century, until the advent of the molecular biology revolution, the generation of shape (morphogenesis) was explained in mechanical terms [1], [2]. Developmental genetics provided important information about the genes involved in the morphogenesis of different organs and appendages, but it did not advance our knowledge of how shape is generated and controlled [3], [4].

To clarify this process, several models have been used [5]–[8]. For instance, skin appendages including the mammary gland develop through complex reciprocal interactions between mesenchyme and epithelium [9], [10]. The latter is represented in the ducts of the breast, and the mesenchyme corresponds to the surrounding matrix and stromal cells. Ducts are the main epithelial structures present in the resting mammary gland. Acini, the structures where milk is produced, develop during pregnancy and persist during nursing. Ducts are the conduits through which milk is delivered. At the end of lactation, the gland undergoes remodeling; acini disappear and ducts remain. Cancer typically develops within ductal structures [11].

*In vitro* 3D culture models facilitate the examination of the factors and events underlying shape determination [12]. Manipulation of the matrix composition results in the generation of the two main epithelial structures, namely elongated ducts and rounded acini [13], [14]. Using this model, we aimed at determining the contribution of local and bulk mechanical properties to the formation of acinar and tubular structures.

A physical component that influences mammary gland morphogenesis is matrix stiffness. In 3D collagen gels, the formation of normal acini requires conditions that simulate the elastic modulus of normal mammary gland tissue [15]. In previous studies, extracellular matrix composition, as well as the presence or absence of fibroblasts, was manipulated to shift the proportion of ducts and acini [13]. The heterogeneous distribution of acini and ducts within the same gel suggests that the local properties of the matrix determine shape. Furthermore, when collagen fibers are aligned by uniaxial tension, tubular structures grow following the direction of the aligned fibers [16]. In addition, morphological changes due to hormone action are accompanied by distinct patterns of collagen organization [17]. Collectively these studies highlight the importance of collagen fiber organization as a local determinant of epithelial shape.

Based on previous work, we hypothesized that a random orientation of collagen fibers was a major determinant in the formation of acinar structures. Conversely, fibers aligned in a prefered direction facilitated the formation of tubular structures. To test this hypothesis, we seeded normal human breast MCF10A cells under conditions where they developed into either acini, ducts or both types of structures. We explored the contribution of matrix rigidity and collagen fiber organization to the shaping of the mammary epithelium through a combination of rheology and quantitative time lapse microscopy. Previously, we have shown that after 3 days in culture, the shape of the epithelial structure was already determined [13]. In the current study, we examined epithelial cell and collagen matrix organization at much earlier time points immediately following single cell seeding. We observed cell motility and collagen fiber remodeling at various distances from the epithelial structures throughout 5 days of growth. Using quantitative collagen analysis, we identified changes in collagen alignment in gels composed of a constant concentration of collagen type I and increasing concentrations of reconstituted basement membrane (Matrigel) and correlated this to the type of structure formed. Furthermore, scanning electron microscopy (SEM) was used to visualize the ultrastructural characteristics of matrices surrounding epithelial structures. This study provides insights into the initial stages of glandular tissue formation and the complex relationships between epithelial breast cells and extracellular matrix organization.

## Results

### Matrigel concentration modulates epithelial morphogenesis

**Figure 1:**
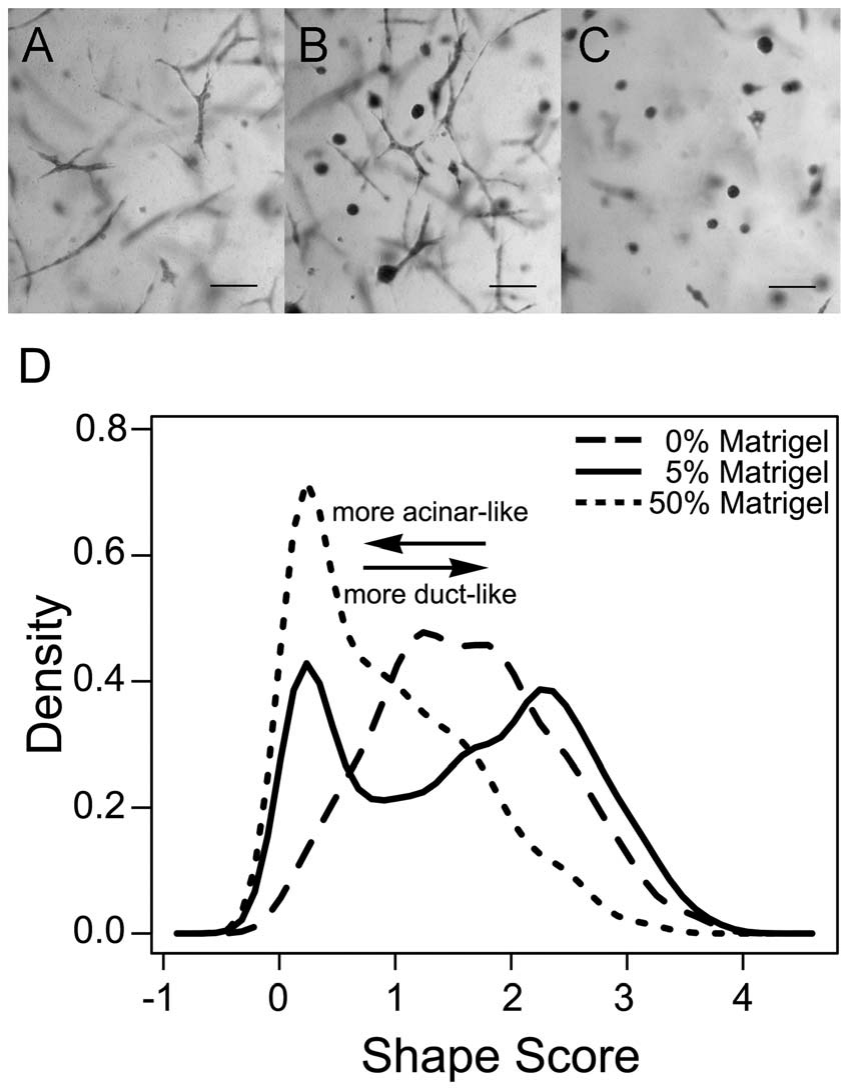
Matrix composition affects the proportion of ducts and acini. Brightfield images of (A) 0%, (B) 5% and (C) 50% Matrigel Carmine-stained whole-mounted gels. Scale bar, 100 μm. (D) Graph comparing the normalized abundance (density) of epithelial structures of different shape score (n = 3 gels).

To allow for high resolution live cell imaging, 3D cultures of MCF10A cells were grown in glass-bottom multiwell plates. This configuration produced a similar distribution of structures to those previously described [13]. At five days after seeding, the distribution of epithelial structures in the gels was largely uniform (Figure 1A, B and C) with the exception of their extreme periphery where ducts organized parallel to the borders of the gel (data not shown). Confocal stacks were acquired in a systematic random sampling to analyze the shape of epithelial structures in each condition. As explained in the experimental procedures, the shape score of each epithelial structure was used to distinguish ducts from acini. Ducts have a higher shape score than acini by definition. As shown in Figure 1D, in 50% Matrigel, the structures formed were more acinar - like, while in 0% Matrigel most of the structures were more duct - like according to the median of shape score (p<10^−15^). In 5% Matrigel, the structures formed were distributed as two distinct populations, which correspond to acini and ducts, as shown by the bimodal distribution in the graph in Figure 1D (p = 9×10^−5^). This bimodality demonstrates the fact that our shape score (partially) segregates acini and ducts. Additionally, in 5% Matrigel a significant difference in the variance of the shape score was observed when compared to 0 and 50% Matrigel (p = 2×10^−8^ and p = 4×10^−12^ respectively).

### Cell proliferation rates do not explain morphological changes due to the presence of Matrigel

Proliferation curves were performed to investigate whether differences in morphogenesis could be attributed to a change in cell proliferation rates caused by the addition of Matrigel. While proliferation rates were significantly lower in 0% Matrigel (p = 0.006 and p = 0.03 against 5% and 50% Matrigel respectively), the rates did not significantly differ between 5% and 50% (p = 0.44) (Figure S1). Therefore, cell proliferation changes did not correlate with the differences in the distribution of shapes between these two formulations.

### Morphogenesis is not significantly affected by bulk gel stiffness

**Figure 2:**
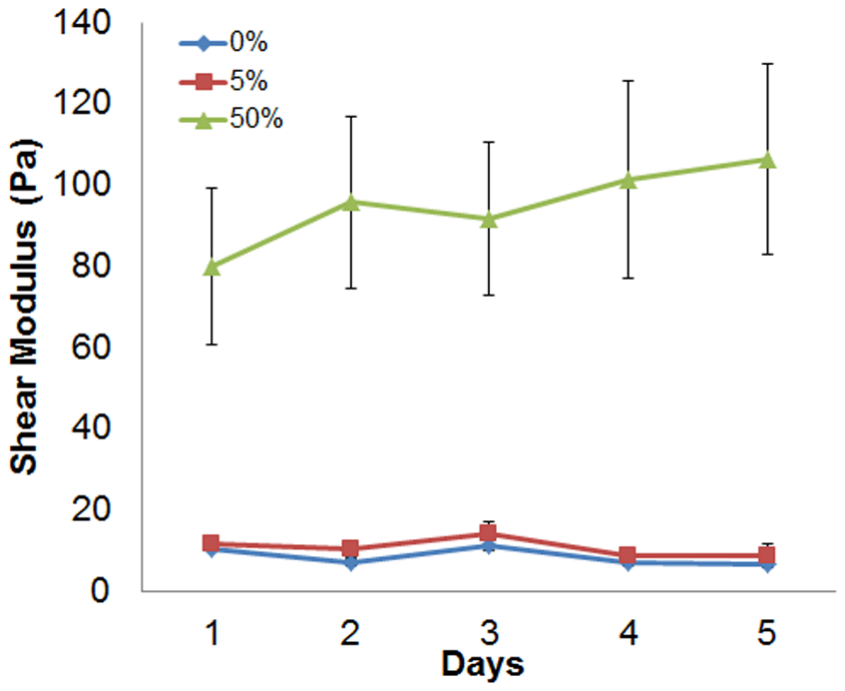
Shear moduli in 0%, 5% and 50% Matrigel over days 1–5. Data are represented as mean +/− SD (n = 4 gels).

To assess the potential correlation between bulk gel stiffness and morphology with the addition of Matrigel, the shear modulus of the gel was measured using a rheometer (Figure 2). No significant difference was detected between gels containing 0% and 5% Matrigel (p = 0.17). However, an 8-fold increase in the shear modulus was observed as the Matrigel concentration increased from 0% to 50% (*p*<0.001). These results indicate that changes in bulk stiffness do not explain the existence of two populations of epithelial structures in 5% compared to the single population in 0% Matrigel.

### The structural characteristics of Matrigel impair the ability of epithelial cells to interact with collagen fibers

**Figure 3:**
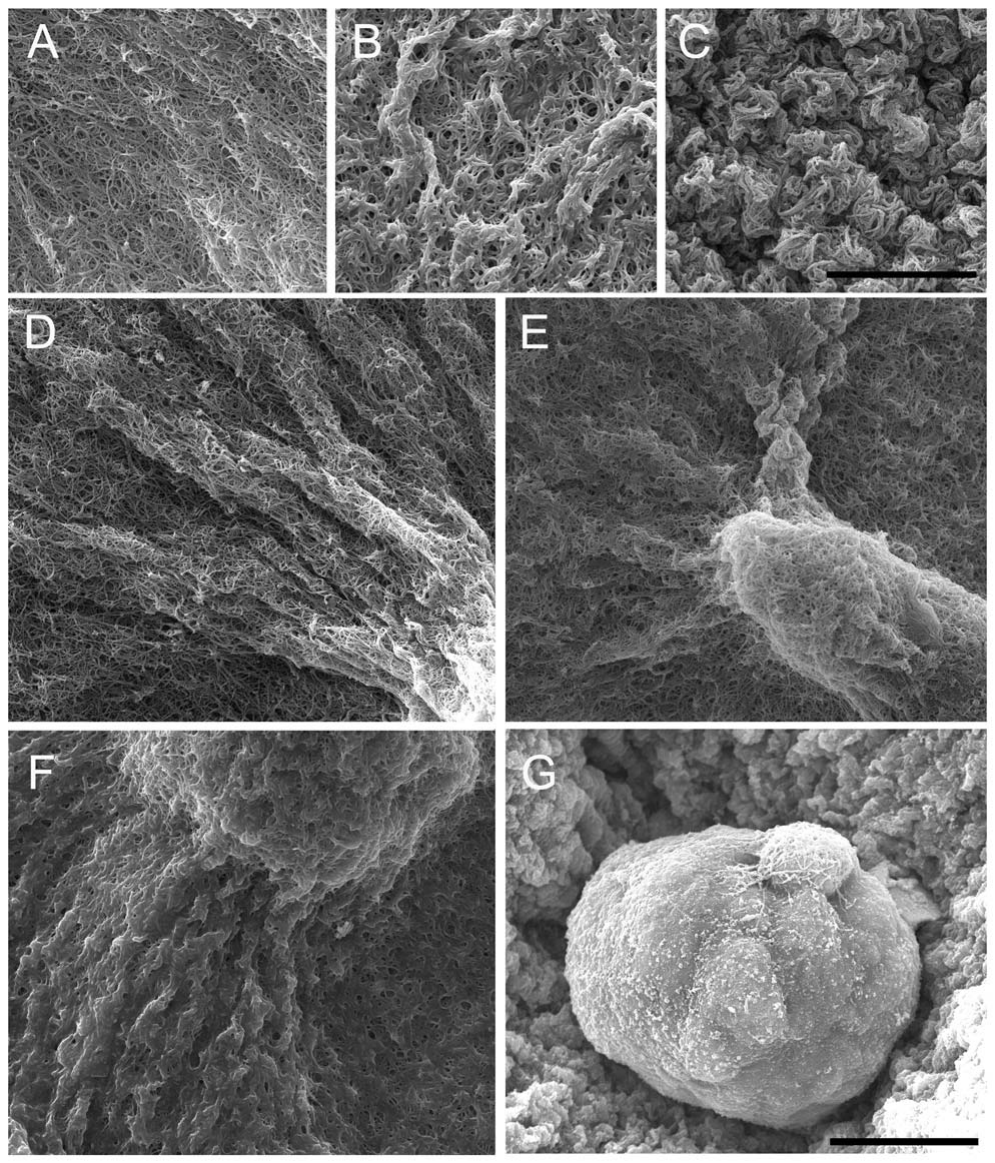
SEM images of epithelial structures and their matrix at day-5. (A) Collagen fibers are clearly distinguished in a collagen-only matrix. (B) The addition of 5% Matrigel results in a globular rather than (A) fibrilar matrix. (C) The globular matrix is more compact in 50% Matrigel. (D) Tubular extensions are observed at the tip of a duct in a collagen-only matrix. (E) Ductal and (F) acinar structures in 5% Matrigel; Matrigel forms a localized coating in areas surrounding the acinus. (G) An acinus grown in 50% Matrigel; collagen fibers are not visible. Scale bar, 10 μm in A to C and 15 μm in D to G.

Scanning electron microscopy (SEM) revealed that the addition of Matrigel changed the organization of collagen fibers (Figure 3). The mesh-like structure of a collagen-only matrix differed from the globular organization of Matrigel-containing matrices (Figure 3A to C). In fact, the average fiber diameter in the collagen-only matrix was 94 ± 29 nm (n = 100), while the presence of Matrigel prevented accurate diameter measurements. In 0% Matrigel, a porous fiber network (Figure 3A) was clearly observed as shown in the leading edge of a duct in Figure 3D. Matrigel was distributed in an heterogeneous fashion in 5% Matrigel. In such gels, cells extended protrusions in areas where Matrigel did not mask collagen (Figure 3E). In contrast, when Matrigel masked collagen, neither pores nor collagen fibers were discerned. This arrangement was characteristic of areas proximate to acini in 5% Matrigel (Figure 3F). In 50% Matrigel, the cells had limited interaction with the network of collagen fibers suggesting that the globular structure of Matrigel creates a barrier that inhibits the extension of cell protrusions into the matrix (Figure 3G). The interaction between epithelial cells and collagen fibers modulates epithelial morphology.

### Changes in collagen fiber organization became apparent early after seeding

The spatiotemporal patterns of collagen organization during mammary epithelial morphogenesis were characterized and quantified during the first 12 hours after seeding. Reflectance confocal microscopy (RCM) was used to characterize collagen organization, while the simultaneous collection of brightfield images enabled the isolation and quantification of epithelial structure morphology (Figure S2).

**Figure 4:**
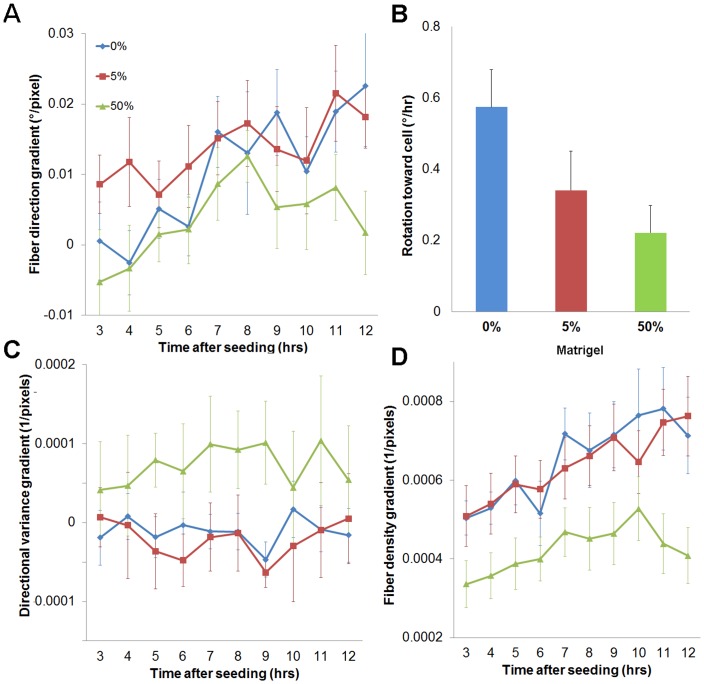
Early changes in collagen spatial organization. (A) The fiber direction gradient increases from the initial time point, suggesting fibers closer to the cell structure become increasingly oriented perpendicular to the cell surface over time. Significant differences relative to the initial time point were only detected in 0% Matrigel at 9, 11, and 12 hrs (p = 0.0001). (B) The rate of fiber rotation over the first 12 hours of culture in regions 40 μm from the cell surface was significantly faster in 0% Matrigel compared to 50%. (C) No significant changes in the directional variance gradient were observed over the first 12 hours indicating a lack of local anisotropy developing within the fiber network at the initial stages following seeding. (D) The spatial gradient of fiber density significantly increased over time in all groups, indicating some level of cell-mediated gel compaction regardless of Matrigel composition. Data are represented as mean +/− SD.

No significant changes in the 2D morphology of epithelial structures as measured by form factor (i.e. the normalized ratio of structure area to perimeter) were detected in any conditions in the series of images taken 3–12 hrs after cell seeding. However, a variety of differences in collagen organization with respect to the orientation and distance of the nearest cell surface were detected (Figure 4). Over time, the mean collagen fiber direction within the first 40 μm from the cells rotated to become more perpendicular to the nearest cell surface in all conditions, with significant differences initially detected at 7 hrs post-seeding relative to the initial imaging time point (p≤0.0315) (Figure 4B). The change in fiber orientation was also computed as a gradient with respect to distance from the cell structure; this orientation gradient increased with time. The average fiber orientation became more perpendicular to the cell surface in regions closer to the structure at most time points, particularly in 0% and 5% Matrigel (Figure 4A). However, significant changes in this fiber direction gradient from the initial time point were detectable only in 0% Matrigel at 9, 11, and 12 hrs (p≤0.0001) (Figure 4A). Additionally, the average rate of fiber rotation over the first 12 hrs in 0% Matrigel was significantly greater than in 50% (p = 0.0495) (Figure 4B). Collectively, these outcomes indicate that fiber orientation changes more rapidly in the absence of Matrigel and that early changes in fiber orientation may be associated with later changes in the morphology of the structure.

The local variance in fiber direction and fiber density was also quantified during the first 12 hours. Directional variance did not significantly alter with respect to time or distance from the cells (Figure 4C), indicating that the strength of alignment in the mean direction within local regions did not consistently change at these initial time points. However, a variety of fiber density measurements yielded significant changes over the first 12 hours. A positive fiber density gradient with respect to distance from the cell surface indicated that more fibers were located in regions closer to the cells for all conditions at all time points (Figure 4D). Furthermore, this positive fiber density gradient increased over time in all conditions with significant differences (relative to the first time point) initially observed at 9 hrs in 0% (p<0.0001), 7 hrs in 5% (p<0.0001), and 5 hrs in 50% Matrigel (p = 0.0002) (Figure 4D). Within the first 40 μm from the cell surface, a greater standard deviation in fiber density values was evident over time (Figure 4), suggesting increasing heterogeneity in the gel organization. Collectively, these quantitative fiber measurements within the first 12 hrs of seeding suggest that distinct patterns of fiber reorganization occur among gel compositions yielding different epithelial morphologies. This initial phase of collagen fiber remodeling occured prior to any detectable change in morphology of the epithelial structures, suggesting that local collagen organization plays a critical role in guiding morphogenesis.

### Time lapse microscopy allows for the visualization of morphogenesis and concomitant changes in collagen organization

**Figure 5:**
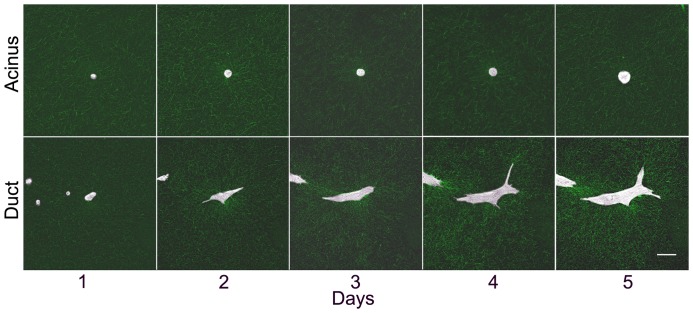
Time-course of epithelial morphogenesis. Still images at a single position taken at day 1 (3 hours after seeding), 2 (27 hrs), 3 (51 hrs), 4 (75 hrs) and 5 (99 hrs) showing the formation of an acinus in 50% Matrigel (top row) and a duct in 0% Matrigel (bottom row). Brightfield and RCM images showing the collagen fibers are overlaid. Scale bar, 50 μm.

Movies of individual cells (n = 34) were produced to visualize the dynamic morphological changes occurring in the presence and absence of Matrigel. Based on the qualitative examination of each epithelial structure as ductal (length ≥ 2*width) or acinar (length ≈ width) at day 5, we found 91% of cells ultimately formed ductal structures in 0% Matrigel. In 5% Matrigel, 70% of the cells formed ducts and 30% formed acini, while almost all structures formed acini in 50% Matrigel (77%); this is consistent with the whole mount analysis (Figure 1). The proportion of acini/ducts was significantly different between 0% and 5% and between 0% and 50% Matrigel (p = 1.93×10^−2^ and p = 8.63×10^−8^, respectively). Cellular protrusions were identified and measured in all groups during the first 12 hours of imaging; they were significantly longer in 0% Matrigel than in 50% (p = 0.017) with an overall average over time of 11.62±7.96 μm and 7.12±3.61 μm, respectively. No significant differences were observed between 0% and 5% Matrigel and between 5% and 50% Matrigel. Over the course of five days, ductal morphology continued to evolve in 0% and 5% Matrigel from dynamic small protrusions to a permanently elongated shape, while structures in 50% Matrigel remained primarily spherical (Figure 5 and Movie S1). Although a slight increase in fiber density was observed over time around acinar structures developing in 50% Matrigel, no strong fiber alignment in any direction was detectable. In contrast, structures with a duct-like morphology demonstrated strong fiber alignment primarily surrounding cell protrusions with an increased tendency of neighboring cells to join together (Figure 5 and Movie S2).

Ductal structures had a maximum of 4–5 cells lying in parallel at the widest point indicating inhibition to lateral growth. Matrix degradation, as inferred by the absence of RCM signal, was commonly observed in 0 and 5% Matrigel in areas where cells had temporarily shifted position; as during mitotic rounding up or when moving closer to a neighbouring structure. Occasionaly, neighbouring cells could move into an area previously degraded but this was not a prerequisite for motion (Figure S3). During ductal formation, some cells detached from the main structure before re-joining later (Movie S2).

**Figure 6:**
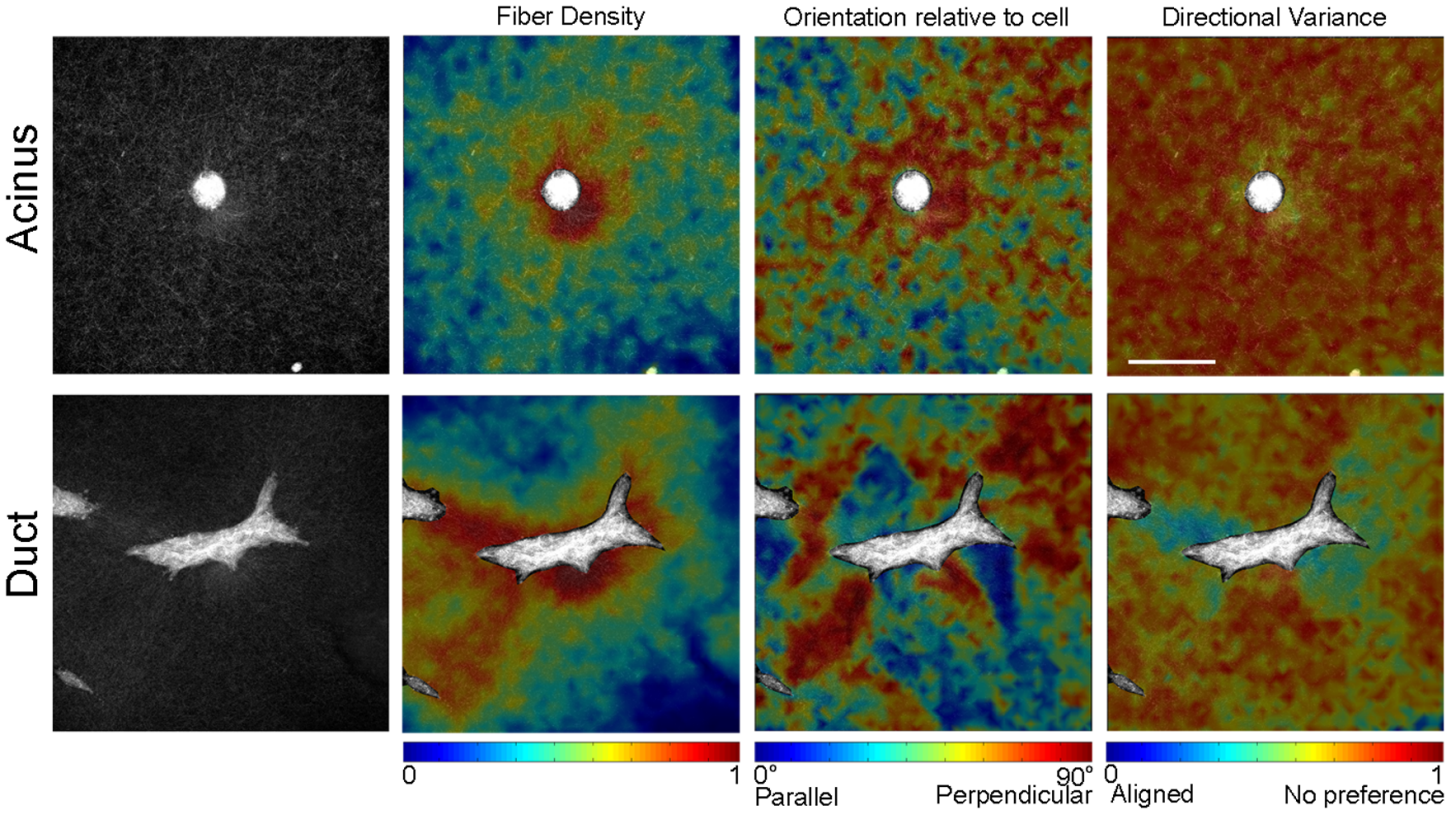
Patterns of collagen fiber organization. Representative fiber maps of an acinus (top) and a branching duct (bottom) at day 5. Scale bar, 50 μm.

Quantitative analysis of collagen fiber organization suggested very different mechanical microenvironments surrounding acini and branching ductal structures. Both acini and ducts demonstrated greater fiber density closer to the epithelium, but the local orientation of the fibers was substantially different. The mean fiber direction surrounding acini was perpendicular to the nearest cell surface; however, the strength of alignment in this mean direction was relatively weak as indicated by the high directional variance (Figure 6). Conversely, elongated and branching ducts exhibited regions of fiber orientation that alternated from being parallel to perpendicular to the cell surface. Substantial changes in directional variance were found surrounding these structures with particularly low values in dense regions of fibers oriented perpendicular to the cell surface (Figure 6). These collagen measurements, demonstrating strong local fiber alignment in discrete regions surrounding the structure, suggested an anisotropic distribution of forces on the collagen fiber network.

### F-actin alignment with collagen fibers

**Figure 7:**
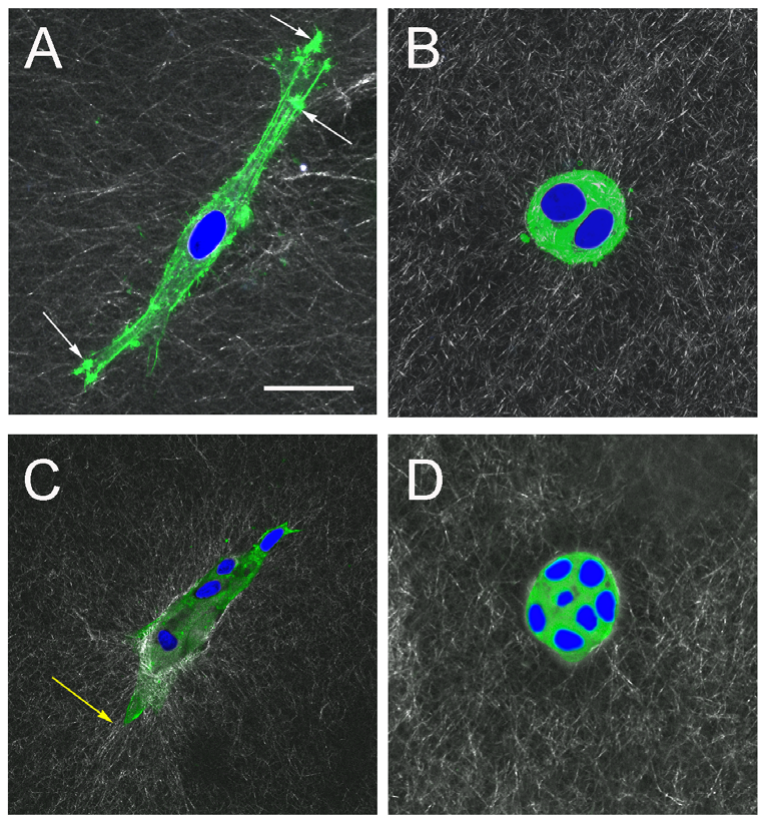
F-actin organization in epithelial cells forming acinar and ductal structures. (A) and (B) are images at day-3 and (C) and (D) at day-5. Nuclei are stained with DAPI. (A) Elongated cell in 0% Matrigel. The presence of localized areas of actin particularly at the leading edges indicates actin-associated adhesion with the ECM (white arrows). (B) Rounded cells in 50% Matrigel. Few filopodia are observed as the majority of actin-staining originates from the cell cortex. (C) Duct in 0% Matrigel. Co-alignment between actin and extracellular collagen fibers is evident (yellow arrow). (D) Acinus in 50% Matrigel, as in (B) the majority of staining originates from the cell cortex and there are no filopodia. Scale bar, 25 μm.

To determine whether changes in the surrounding collagen fiber organization were associated with cell mediated traction forces, gels were stained for F-actin at day 3 and 5 post-seeding (Figure 7). Co-alignment of F-actin and collagen fibers was evident in ducts at both time points (Figures 7A and C). Particularly high densities of staining were visualized at the leading edges of ductal structures and at early branching points. These findings indicated that actin - mediated transmission of traction forces was likely in these areas as co-alignment is strongest. In contrast, the distribution of F-actin in acinar structures at day 3 and 5 in both 5 and 50% Matrigel showed little alignment with that of fibers in the surrounding matrix; most staining was homogeneously distributed in the cytoplasm and more intense in the cell cortex (Figures 7B and D).

### Locally aligned collagen fibers lead to branching ducts

**Figure 8:**
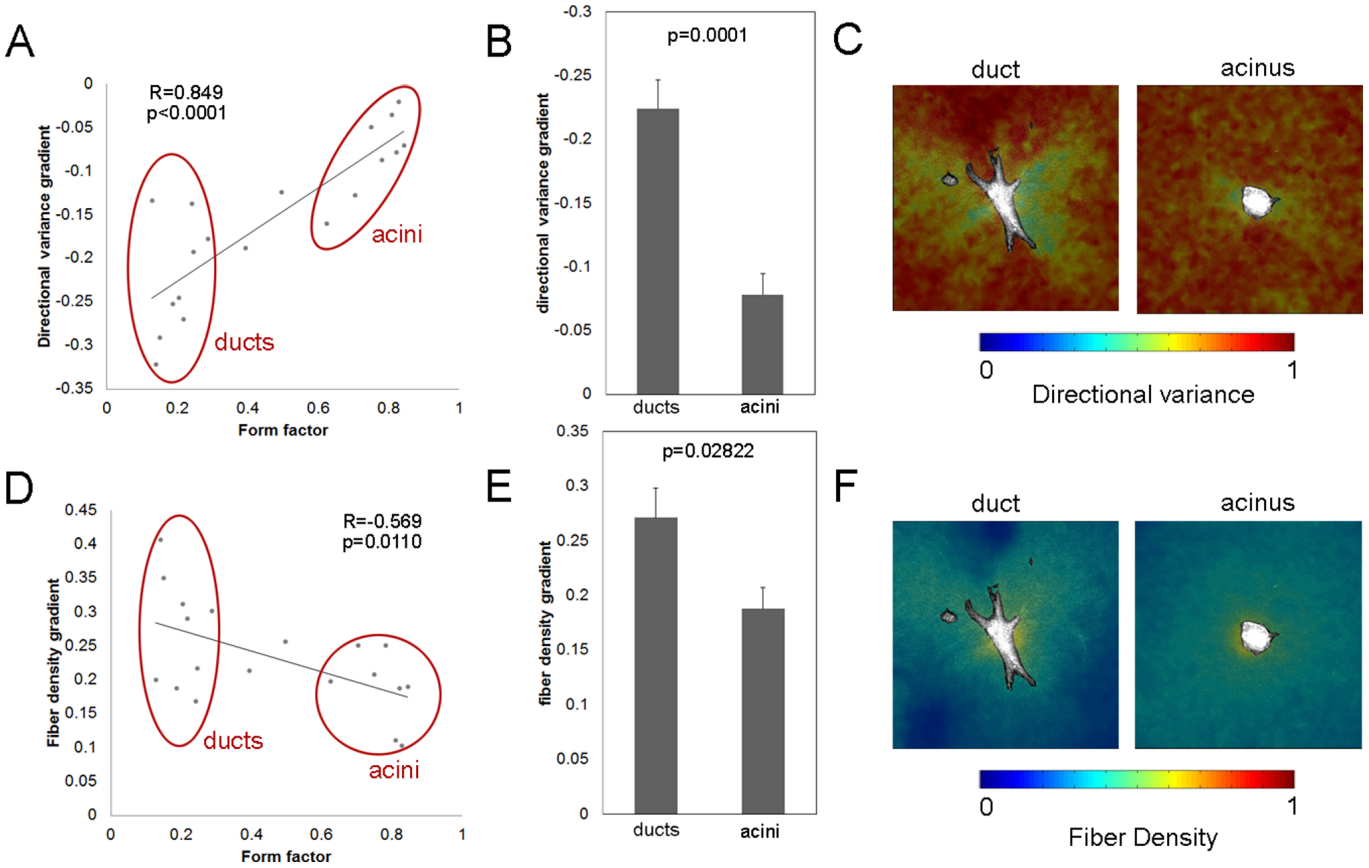
Differences in fiber organization between ducts and acini formed within 5% Matrigel at day 5. (A) The directional variance gradient of the collagen fibers is correlated with the form factor of the epithelial structure. (B) A significantly greater directional variance gradient surrounds ducts compared to acini. (C) Representative maps of directional variance indicate substantial variability surrounding ducts. (D) Fiber density gradient is also correlated with form factor. (E) Ducts have a greater fiber density gradient than acini. Data are represented as mean +/− SD. (F) Representative maps of fiber density surrounding the structures demonstrate a greater fiber density gradient during duct formation compared to acini.

In order to explore the relationship between collagen fiber organization and morphogenesis, while isolating effects related to different gel compositions, a quantitative analysis was performed on the different structures formed by day 5 in 5% Matrigel. As in the quantitative analysis of cell morphology during the first 12 hours, form factor was used to characterize the shape of structures at day 5. Epithelial structure form factor was strongly correlated with the directional variance gradient of the collagen fibers (R = 0.849; p<0.0001), indicating that more rounded structures were produced when collagen alignment was mostly isotropic and that elongated structures were produced when collagen fibers were strongly aligned near the cells (Figure 8A). A significantly greater directional variance gradient was detected in ducts (form factor <0.3) compared to acini (form factor >0.6) (p = 0.0001) (Figure 8B). Furthermore, ducts showed significant variability in directional variance immediately surrounding the cells (Figure 8C). In fact, the standard deviation of directional variance within the first 40 μm from the epithelial surface was also significantly correlated with form factor (R = 0.5636; p = 0.0120). These regions of highly aligned collagen fibers surrounding the ductal structure would suggest increased tensile load and an anisotropic mechanical environment.

In addition to the defined relationship between structure form factor and collagen directional variance at day 5, the fiber density gradient was significantly associated with differences in morphology (R = 0.569; p = 0.0110) (Figure 8D). Specifically, a higher fiber density gradient was associated with the lower form factor values (Figure 8D), and a signficantly higher fiber density gradient surrounding ducts was observed compared to acini (p = 0.0282) (Figure 8E and F). In summary, these quantitative measurements of epithelial morphology and collagen organization indicated that the formation of ducts was associated with heterogenous fiber organization and that a complex, anisotropic mechanical environment exists surrounding these structures 5 days after seeding.

## Discussion

Using a combination of time-lapse microscopy and static measurements of tissue ultrastructure and bulk material properties, we have explored factors that may influence the probability that a single epithelial cell will ultimately form an acinus or a duct in a 3D culture model. Specifically, we found that changes in collagen fiber organization can be detected within hours of cell seeding, and that ultimately a strong relationship exists between collagen fiber organization and the development of distinct structures. These dynamic changes in fiber organization over time suggest a corresponding change in the mechanical properties of the extracellular matrix (ECM), which has long been considered a main determinant of epithelial shape. Previous work at discrete time points has suggested that increased bulk rigidity hinders the formation of acini [15], and that fiber organization influences the organization of epithelial structures [13], [14], [16], [18]. This outcome happened even when the matrix composition was held constant and thus, changes in epithelial shape cannot be attributed to matrix composition [16]. Furthermore, the distribution of epithelial structures was similar in mixed gels (1 mg/ml collagen I containing 50% Matrigel) regardless of whether the gels contained Matrigel or Matrigel reduced in growth factors (unpublished data). This finding supports the idea that the biochemical composition of standard Matrigel in terms of “growth factors” does not seem to play a significant role in epithelial organization. Using our previously described 3D model, wherein the proportion of acinar and ductal structures could be modified by changing the concentration of Matrigel in the ECM, we have now developed image analysis techniques to quantify the differences in collagen fiber kinematics and organization that govern morphogenesis.

Through both qualitative and quantitative evaluations of epithelial morphology and collagen fiber organization from live microscopy images, we have developed a framework for elucidating the sequence of events that drives epithelial morphogenesis. During the first 12 hours following seeding, small cell protrusions were observed at certain time points and most frequently in gels containing collagen only. The increased kinematic activity of these cells coincided with the initial quantitative detection of significant changes in both fiber alignment and density at approximately 7 hours post-seeding (Figure 4 and Figure S2). Collectively, these findings during the first 12 hours suggest that breast epithelial cells extend projections and apply traction forces to the ECM through focal adhesions soon after seeding.

In gels containing only collagen, the matrix easily deforms in response to the cell traction forces. Specifically, when collagen fibers were rotated toward the direction of the inferred traction force, fiber density increased near the cell (Figure 4). Previously, a number of studies have demonstrated that collagen fiber networks reorient in the direction of applied loads [19]–[21]. Because static equilibrium is easily achieved through ECM deformation and realignment, the overall epithelial morphology did not appear to significantly change during the first 12 hours. However, collagen fiber rotation toward the direction of the inferred traction forces over 5 days resulted in strong fiber alignment (i.e. low directional variance) within certain regions of the surrounding ECM (Figure 6 and Figure 8). In these locations with strong fiber alignment, the ECM should be much stiffer in the mean direction of the fibers, and consequently, the epithelial structure will deform in response to the applied traction force. As a result of the increased gel stiffness in the direction of the aligned collagen fibers, ductal structures elongated in the same direction as the collagen fibers and exhibited substantial changes in overall morphology. The alignment of the collagen fibers, and the resulting local anisotropic material properties of the collagen gel, continued to guide cell elongation and migration over time, and ultimately a strong correlation between epithelial morphology and collagen organization was observed among these structures at day 5 (Figure 8).

Epithelial morphology was modulated in this study by altering the steric and mechanical properties of the ECM through the addition of Matrigel. In 0% Matrigel, the development of highly anisotropic collagen fiber organization over time gave rise to an abundance of elongated ductal structures. However, in 50% Matrigel, acini were the most frequent structures present. When viewed by SEM, collagen fibers were distributed in a mesh-like network in 0% Matrigel, while in 50% the fibers curled into globular arrangements ([22] and Figure 3) and individual fibers could not be easily distinguished. The increased stiffness in 50% Matrigel relative to the 5% and 0%, and the masking of the fibers, may have together prevented the collagen fibers from freely rotating during the application of traction forces. This lack of collagen fiber rotation in 50% Matrigel (Figure 4B) may have prevented the formation of an anisotropic mechanical environment capable of facilitating epithelial elongation. Furthermore, the reduced porosity evident in SEM images of 50% Matrigel may have also prevented the extension of cell protrusions into the ECM. Indeed, we observed that in 0% Matrigel cells extended significantly longer protrusions in the first 12 hours after seeding than in 50% Matrigel. This finding is consistent with the fact that vascular cell elongation is impaired in collagen matrices containing a high concentration of Matrigel [23], [24]. Collectively, the steric properties [25] and the mechanical response of the ECM in 50% Matrigel may have attenuated changes in fiber organization observed during the first 12 hours (Figure 4), which prevented the formation of ductal structures.

Although the proportion of acini and ducts was signficantly different between 0% and 50% Matrigel, 5% Matrigel (which contains micro-domains of differing collagen organization) produced a mixed population of these two structures. However, this increasing trend in the proportion of acini with increasing Matrigel concentration (Figure 1) did not match differences in the bulk material properties of the gels (Figure 2). The shear moduli of both 0% and 5% Matrigel were nearly identical and significantly lower than 50%. Nevertheless the rate of collagen fiber orientation toward the epithelial structure during the first 12 hours matched the observed trends in the proportion of ducts and acini at day 5 (Figure 1 and Figure 4B). SEM images demonstrated that the ultrastructure of the matrix in 5% Matrigel was clearly heterogeneous with discrete areas similar to those found in either 0% or 50% Matrigel (Figure 3). Not surprisingly, in 5% Matrigel, ducts were observed in regions with an ultrastructure resembling 0% Matrigel, whereas acini were formed in regions similar to those of 50% Matrigel. The quantitative analysis of structures formed in 5% Matrigel confirmed the existence of a strong correlation between collagen fiber organization and the shape of the epithelial structures (Figure 8). The ECM surrounding acini showed weak collagen fiber alignment, while ductal and branching structures displayed strong local fiber alignment in discrete regions (Figure 8C). These results further indicate that anisotropic material properties produced through alignment of the collagen fiber network are associated with the formation of tubular and branching structures, while random fiber orientation distributions correspond to the formation of rounded structures.

F-actin organization in ducts and acini contribute to the differences in fiber organization between the two types of structures. The actin-mediated protrusions in which F-actin coaligns with collagen fibers are an indication of active force transmission between the ECM and the cells. MCF10A cells appear to pull collagen fibers toward the structure as evidenced by the increase in the fiber density gradient (Figure 4D). F-actin organization suggests that these traction forces were applied during cell protrusion (Figure 7). In 0% and 5% Matrigel, zones of degraded matrix were also observed in areas previously occupied by cells as they changed shape (Figure S3). The degraded areas are thought to be necessary for cell movement as pericellular proteolysis has been found to enable organization of the fibers with microtracks left behind which allow other cells to freely migrate along [26]. Cells were observed moving in directions initially probed by protrusions, indicating that ECM adhesions drive cells forward and that degradation is initially unnecessary until the main body of the cell occupies a new area. Local accumulation of Matrigel around cells in 5%, and to a greater extent in 50% Matrigel, may be sufficient to inhibit the elongation and formation of ductal structures. Acinar structures showed a high density of actin at the cell cortex, probably corresponding to the rotational movement observed during acinar formation [27], [28].

The ability to observe changes in fiber organization in real time for up to five days was made possible by the use of confocal reflection microscopy. An alternative technique, second harmonic generation (SHG) imaging, has been used previously as a non-destructive, label-free technique to characterize collagen fiber organization. SHG imaging, particularly in the context of tumor invasion, has similarly suggested that cell migration is substantially enhanced when collagen fibers are aligned perpendicular to the cell surface [29]–[31]. Additionally, SHG imaging of angiogenic sprouting has shown that collagen aligns in the direction of sprout protrusions and that sprout outgrowth occurs along the direction of the collagen fibers [32], [33]. These observations match the cell-matrix interactions observed at later time points in our RCM study of breast epithelial morphogenesis. Despite the emerging utility of SHG imaging, the laser power required for imaging of the collagen-Matrigel ECM models used in our study has been found to be phototoxic for time-lapse imaging over the course of lengthy periods of observation. RCM using our microscope setup enabled a high degree of 4 dimensional resolution without indications of photodamage. Regardless of the imaging modality used, the analysis methods developed in this study to quantify fiber organization can be applied to a wide range of microscopy approaches.

The distinct fiber organization surrounding acini and branching ducts imply very different mechanical microenvironments. The tension applied by cells to the collagen fibers can be transmitted over a long range [34]; this enables epithelial structures to merge over time, a process that results in the formation of a ductal network after two weeks [13], [16]. The formation of the ductal network has some parallels with the formation of microvascular networks in collagen gels [24], [32], [33]. The present study suggests that force transmission through the collagen network is a main determinant of ductal formation and that the quantitative data presented in this paper could serve as a basis for computational modelling of the dynamics of morphogenesis. A number of computational models have already been developed to predict fiber network kinematics and ECM deformation in response to external forces [20], [21], [35]. Collectively, an integration of computational modeling and quantitative live cell microscopy will be necessary to further elucidate the complex interplay between cells and the surrounding matrix during epithelial morphogenesis.

## Conclusions

Through a combination of live cell imaging and mechanical and ultrastructural assessments at static time points, we have elucidated the role of collagen fiber organization and ECM mechanical properties on epithelial morphogenesis. Quantification of spatiotemporal patterns of collagen fiber organization during the five days following cell seeding demonstrated a strong relationship between epithelial cell morphology and collagen fiber alignment. At early time points, cells organize the collagen fibers most likely through the application of traction forces. In turn, at later time points, this differential fiber organization determines epithelial morphology. By altering the composition of 3D matrices, we demonstrated the ability to control epithelial morphology and, ultimately, the formation of ductal or acinar structures.

## Methods

### Cell culture reagents

Hydrocortisone, cholera toxin, and insulin were purchased from Sigma-Aldrich (UK). Dulbecco's modified Eagle's medium/F12 (DMEM/F12), penicillin–streptomycin solution, L-Glutamine and trypsin were obtained from Gibco/Invitrogen (UK). HyClone equine serum was purchased from Davidson and Hardy (UK). Epidermal growth factor (EGF) was obtained from VWR (UK).

### Cell maintenance

The human breast epithelial MCF10A cell line was provided by the laboratory of Dr. Senthil Muthuswamy [36]. MCF10A cells were grown in DMEM/F12 containing phenol red, 5% equine serum, 20 ng/ml EGF, 0.5 μg/ml hydrocortisone, 0.1 μg/ml cholera toxin, 10 μg/ml insulin, and 1% penicillin–streptomycin solution. Cells were incubated at 37°C in 6% CO_2_/94% air and maintained in plastic cell culture flasks (Corning, NY, USA). Cells were used between passages 40–49.

### 3D cultures

Bovine type-I collagen, at a concentration of 5 mg/ml, was purchased from Organogenesis (MA, USA). It was diluted with 0.05% v/v acetic acid (Sigma-Aldrich, UK) yielding a stock solution of 1.4 mg/ml, and neutralized with 75 mg/ml sodium bicarbonate [16]. The final collagen concentration in all gels was 1.0 mg/ml in Eagle's medium (Lonza, UK) containing 200 mM L-glutamine and 10% FBS (HyClone FBS, Davidson and Hardy, UK). Cells were detached by trypsin treatment and counted using a NucleoCounter NC-100 (ChemoMetec, Denmark) before seeding in the collagen mixture at 50,000 cells/ml. To each well 500 μl of the cell-collagen mixture was carefully pipetted into a glass-bottomed 6 well plate (No. 1.5, 14 mm Mattek, USA). The gels were allowed to solidify for 30 min at 37°C before adding 1.5 ml of cell maintenance culture medium into each well. Cultures were maintained for 5 days, and the medium was changed every 2 days.

Gels containing 5% v/v Matrigel (BD Biosciences, UK) and 1 mg/ml collagen were prepared in a similar manner to that described above, except that the collagen stock was diluted with 0.05% acetic acid to a working solution of 1.485 mg/ml before adding an appropriate volume of Matrigel to give a 5% v/v Matrigel concentration. Gels containing 50% v/v Matrigel and 1 mg/ml collagen required a collagen working solution of 2.8 mg/ml, and the addition of an appropriate volume of Matrigel to give a 50% v/v Matrigel concentration.

### Whole mount analysis

After 5 days, gels were mounted on slides, fixed and stained with Carmine [16]. Confocal stacks of the whole mounted gels were acquired using an automated systematic random sampling method (Leica SP5 software). ImageJ plugins, 3D Object counter [37] and 3D ROI manager [38] were used for shape analysis of the epithelial structures. To analyze the shape of the structures, we chose the approach of fitting the structure into an ellipsoid. The shape descriptors Elon1 and RatioVolEllipsoid were chosen to analyze the types of structures formed: acini or ducts [38]. Briefly, Elon1 is the ratio between the major axis and the middle axis of the 3D fitted ellipsoid and RatioVolEllipsoid is the ratio between the volume of the 3D structure and the 3D fitted ellipsoid. Structures smaller than 300 voxels were excluded from the analysis. In general, for structures that are elongated and not branching, the corresponding ellipsoid has a major axis far larger than the middle axis, and so Elon1 is substantially greater than 1. If the structure is spherical as in the case of an acinus, Elon1 is approximately 1. However, a branching duct does not fit well into an ellipsoid, and this can result in low or intermediate Elon1 values. In order to resolve this, we used the ratio *r* of the volume of the ellipsoid by the volume of the structure (1/RatioVolEllipsoid). Non-branching ducts have a high Elon1 and low r, while branching ducts have a low or intermediate Elon1 and high r. Contrariwise, acini have low Elon1 and low r. In order to distinguish acini and ducts we defined a “shape score” as log(Elon1*r). We computed the normalized histogram of the diverse shape scores for epithelial structures ranging from acini to ducts.

### Cell Proliferation

Cells were dissociated from gels for counting every 24 hrs over a five-day period through a two-step process. Gels were initially treated with Dispase (BD Biosciences, UK) according to manufacturer guidelines followed by collagenase treatment [17]. Cells were counted as described above.

### Rheology

Evaluation of viscoelastic properties was performed using a Kinexus Pro Rheometer (Malvern, UK). Using Parallel-plate geometry (20 mm×65 mm), gels were placed onto the lower plate of the rheometer, while the higher plate oscillated on the sample at a gap size of 1 mm. For each of the samples, small deformation linearity (linear viscoelastic region) was identified via strain measurement under the application of controlled stress sweeps. Optimum conditions such as shear strain at 1.0% of the angular single frequency (1 Hz) were selected. All measurements were performed at a temperature of 37±0.02°C (n = 4, from two independent experiments). Shear modulus (G′) measurements were derived from G′/G″-frequency (ω) plots produced by single frequency measure. Data was analyzed using proprietary software (rSpace).

### Scanning Electron Microscopy

Gels were transferred to glass containers in 2X Karnovsky's fixative overnight with an equal volume of culture medium. After washing in phosphate buffered saline (PBS), samples were post-fixed in 1% aqueous osmium tetroxide (Agar Scientific, UK) for 1 hour. Following further washing in PBS, samples were dehydrated in graded ethanol solutions of 70, 80 and 90% for 1 hour each. For the final dehydration steps, 1% w/v phosphotungstic acid (Sigma-Aldrich, UK) in 100% ethanol was used for 30 mins followed by another 30 mins in 100% ethanol. Gels were then chemically dried using hexamethyldisilazane (HMDS) (Agar Scientific, UK) in a graded ethanol/HMDS series (1∶3, 1∶1 and 3∶1) before air-drying and mounting on a self-adhesive carbon pad. Gold/palladium was splattered onto the samples to give a coating of ∼70 Å thick. Images were acquired with a Quanta 200 environmental SEM (FEI Company, Netherlands) operated in high vacuum mode. Collagen fiber diameter was measured in three different fields of view using the line measure function in Image J at the mid point of the fiber length.

### Light Microscopy

Live cell-imaging was conducted with a Leica SP5 microscope (Leica-Microsystems, Germany). Cells were maintained at 37°C in an atmosphere containing 6% CO_2_/94% air using a thermostatically controlled heated enclosure with passive humidification and a separate gas mixing unit (Life Imaging Services, Switzerland). Images were collected using a 40×, 1.1 numerical aperture, water immersion objective (Leica-Microsystems, Germany). Immersol W (Zeiss, UK) with a refractive index of 1.334 was used as a water-substitute immersion fluid for long-term imaging. Simultaneous RCM and brightfield images were acquired with separate photomultiplier tubes (PMT) using the Argon 488 nm laser line. The RCM signal was collected between wavelengths of 478–498 nm with a pinhole size of 57 μm. Images were collected with a line scanning speed of 600 Hz at 1024×1024 pixel resolution. A line and frame average of 2 was applied. A total of 34 fields of view per condition were followed by time-lapse microscopy. Representative fields of view were selected from structures formed by single cells. For the quantitative analysis of fiber organization, structures from 0% (n = 12), 5% (n = 11), and 50% (n = 10) Matrigel were analyzed at each hour between the first 3–12 hours following cell seeding. At day 5, morphology and fiber organization were quantified for 19 independent structures in 5% Matrigel.

### F-actin visualization

F-actin was visualized in three- and five-day old gels [17]. Briefly, gels were fixed with paraformaldehyde, permeabilized with Triton X-100 and incubated with 0.14 μM rhodamine-labeled phalloidin (Cytoskeleton Inc, CO, USA). Cell nuclei were stained with DAPI (Sigma Aldrich) before confocal imaging.

### Quantitative analysis of collagen fiber organization

To analyze collagen organization and cell morphology during the first 12 hours after seeding, representative optical sections were selected from the acquired z-stacks at a depth that hemisected the cell/epithelial structure. A 512×512 pixel region centered around the cell of interest was selected for subsequent quantitative analysis. To define epithelial morphology and fiber orientation relative to the nearest cell surface, a binary mask of the epithelial structure was defined from the transmission images. Horizontal and vertical Prewitt edge-finding filters were applied to the transmission image, and a threshold of 25% of the maximal value of the filtered transmission image was applied to define a mask containing the cell/epithelial structure. Each mask was compared to the original image and any discrepancies in the cell boundaries were corrected by manual digitization. Using the binary cell mask, structural morphology was quantified by the form factor (4π*Area/Perimeter^2^) of the structure [17]. To define the distance of every extracellular pixel from the nearest cell surface, the cell mask was incrementally dilated until the entire image was filled by the dilated mask. The sum of these incrementally dilated binary masks provided a means to calculate the distance of each extracellular pixel from the nearest cell surface, and the orientation of the intensity gradient produced by the summed masks corresponded to the orientation of the nearest cell surface (Figure S2).

A previously established image processing algorithm was applied to calculate the orientations of collagen fibers and the nearest cell surface at each pixel by identifying the direction with the minimal amount of variance in intensity surrounding each pixel [39]. Pixel-wise collagen fiber orientation maps were calculated from the RCM images, while the orientation of the nearest cell surface was detected by applying this algorithm to the gradient map generated by summing dilated cell masks as described above.

Local fiber organizational statistics were quantified by discretizing the extracellular space into small triangular elements (each containing approximately 200 pixels), and computing mean fiber direction, directional variance, and fiber density within each regional element. Specifically, a grid of nodes spaced 20 pixels apart within the extracellular regions of the image was produced and a mesh of triangular elements was created through Delaunay triangulation (Figure S2). Within each triangular element, the average fiber orientation relative to the cell, the average distance to the nearest cell surface, fiber directional variance, and fiber density were calculated. To calculate fiber density from RCM images, pixels intensity values were first normalized to account for varying levels of background intensity by calculating the mode of intensity values in the image. Specifically, RCM intensity values at each pixel were subtracted by the mode of the values from that image and a collagen mask was obtained by identifying pixels with background-normalized intensity values that exceeded 2 on an 8-bit scale (0–255). Fiber density within each triangular element was calculated as the proportion of pixels within the element that was part of the collagen mask. The mean fiber direction and directional variance were computed within the element through a directional vector summation approach [40] using only values within the collagen mask regions. The absolute value of the difference in the mean collagen fiber orientation and nearest cell surface orientation was computed for each element with a minimum of 0° corresponding to fiber alignment parallel to the cell surface and a maximum of 90° corresponding to fiber alignment perpendicular to the cell surface.

From each image, means and standard deviations of each fiber measurement were computed from spatial elements within 40 μm from the cell surface. Additionally, gradients in the fiber measurement values with respect to the distance from the nearest cell surface were computed by fitting element-wise scatter plots of each fiber measurement, and their respective distances to the cell surface, to a linear function (Figure S2). Gradients of relative fiber orientation, directional variance, and fiber density were recorded from the slope of these linear functions.

A similar analysis approach was applied to quantifying cell structure morphology and fiber organization at day-5 post-seeding. However, due to increased epithelial structure size and morphological complexity, a single representative depth section could not be selected. Instead, pixel-wise fiber orientation data were processed for each depth slice spanning the structure (18–31 slices). Fiber direction was averaged across the depth slices at each pixel location to obtain a fiber orientation map for further element-wise analysis. RCM intensities were also averaged through the depth slices at each pixel location and an average intensity threshold of 1 was selected to identify pixel locations containing a collagen fiber through the depth. Similarly, epithelial morphology and cell surface orientations at day-5 were computed from an average intensity projection of the Prewitt-filtered transmission images. Correlation coefficients between epithelial morphology and each fiber measurement were recorded. Those structures with a form factor of less than 0.3 were classified as ducts at day 5, while structures with a form factor exceeding 0.6 were classified as acini.

### Statistical analysis

To compare differences in shape score, the Wilcoxon rank test was used. For the analysis of shape score, Hartigans' dip test for unimodality was used to determine whether the distribution departed from unimodality. Levene's test was used to analyze the variance of the average of shape score. Cell proliferation data were analyzed with Cran R by using an analysis of variance (ANOVA) with a linear model. A Chi square test was used to evaluate the differences in the type of structures formed by a single cell tracked by live-imaging for 5 days. The effects of varying Matrigel concentration on gel stiffness and changes over time were evaluated using ANOVA. Regarding the collagen organization analysis, differences in form factor or fiber organization over the first 12 hours following seeding, were assessed through ANOVA. Post-hoc Bonferroni-corrected t-tests were used to compare each time point to the initial time point. Student's t-tests were used to compare quantitative fiber measurements among structures classified as ducts or acini within 5% Matrigel at day-5. Correlations were evaluated with the null hypothesis that R = 0. Signficance for all tests was defined as p<0.05. Results were shown as means with variability reported as standard deviation.

## Supporting Information

Figure S1 **Comparison of cell proliferation rates of MCF10A cells growing in 0%, 5% and 50% Matrigel.** Doubling times were 1.06, 0.86 and 0.89 days for 0% (filled triangle, dashed line), 5% (filled circle, continuous line) and 50% Matrigel (empty circle, dotted line) respectively (r2 = 0.94; 0.99 and 0.98). Each point corresponds to a single gel; lines are regression lines.
(TIF)

Figure S2 **Overview of the image processing steps for quantifying fiber organization.** Transmission and confocal reflectance images were used to isolate cell and fiber structures, respectively. Transmission images were filtered and segmented to define a cell mask, and the orientation relative to the nearest mask surface was defined for each pixel location. Fiber orientation was defined at each pixel as previously described [39], and directional statistics were computed within discrete extracellular regions defined by a triangular element mesh. The gradient of fiber data values with respect to the distance from the cell surface were computed using all extracellular elements, and the average fiber statistics from elements within just the first 40 μm from the cell surface were also computed.
(TIF)

Figure S3 **Collagen degradation during early stage morphogenesis.** Reflection confocal (top row) and brightfield (bottom row) images taken at 8, 9 and 10 hours post-seeding. (A) At 8 hours the cell at the center of the field has degraded an area of collagen (arrow) while the lower cell has a cell protrusion extending towards the upper left. (B) At 9 hours the cell at the center begins to move into the degraded collagen area while the lower cell moves to produce a protrusion pointing to the lower right revealing an area of degradation (arrow). (C) At 10 hours the cell at the center, as well as the one below, now inhabit zones of degradation previously revealed, indicating continuous movement into and out of these zones.
(TIF)

Movie S1 **Formation of an acinus.** Single cells forming an acinus over 5 days in 50% Matrigel. Brightfield images from a confocal z-series are focus stacked and overlaid with green, single - plane, reflection confocal microscopy images to show collagen remodelling. Each frame corresponds to one hour. Still images are depicted in Figure 5.
(MOV)

Movie S2 **Formation of a duct.** Single cells forming a duct over 5 days in 0% Matrigel. Brightfield images from a confocal z-series are focus stacked and overlaid with green, single -plane, reflection confocal microscopy images to show collagen remodelling. Each frame corresponds to one hour. Still images are depicted in Figure 5.
(MOV)
