# Supplementary figures and images for: From Single Cells to Tissues: Interactions between the Matrix and Human Breast Cells in Real Time
Source: PLoS One. 2014 Apr 1;9(4):e93325. doi: 10.1371/journal.pone.0093325 (PMC3972096; doi:10.1371/journal.pone.0093325)

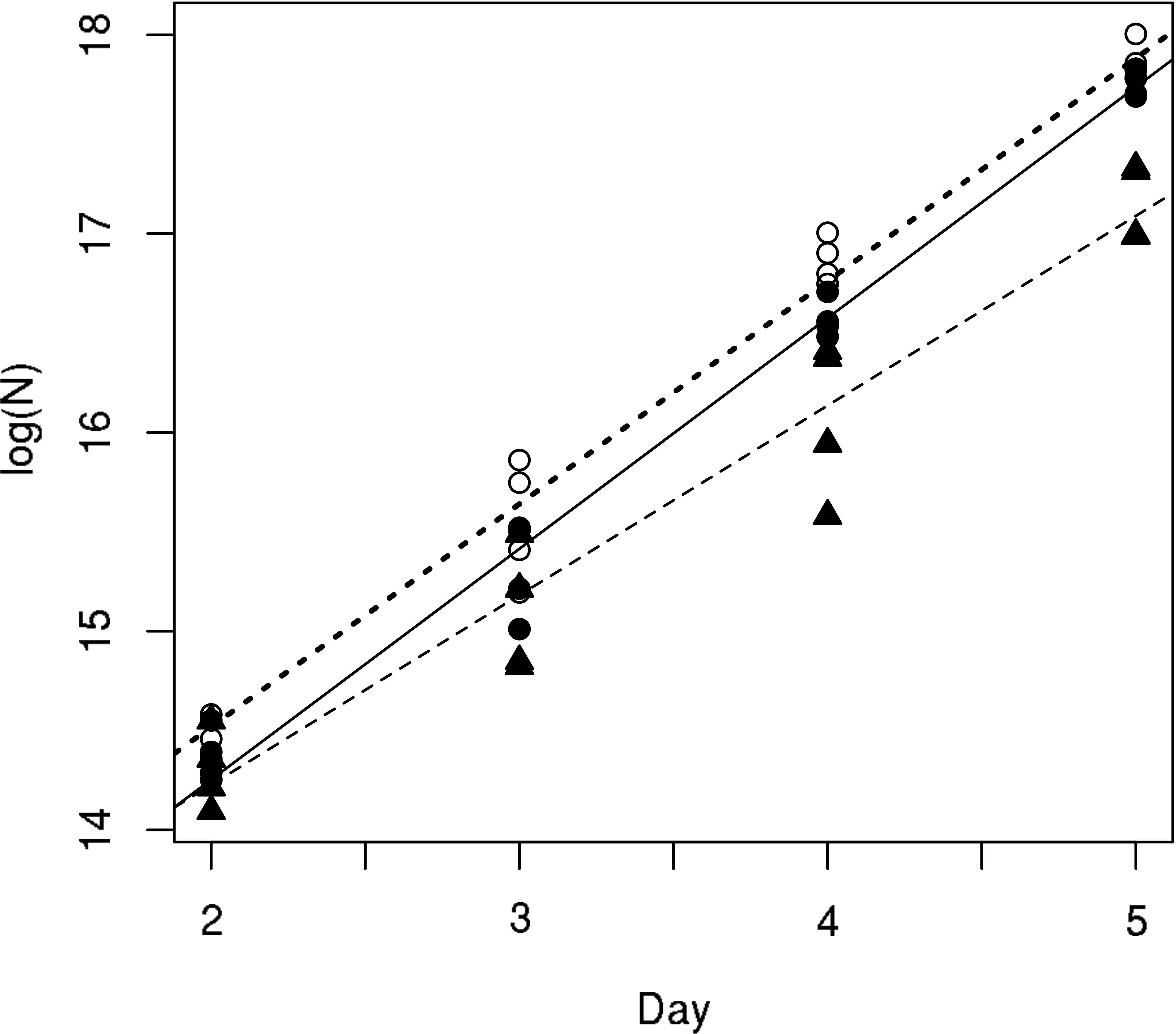

Supplement: Figure S1 — Comparison of cell proliferation rates of MCF10A cells growing in 0%, 5% and 50% Matrigel. Doubling times were 1.06, 0.86 and 0.89 days for 0% (filled triangle, dashed line), 5% (filled circle, continuous line) and 50% Matrigel (empty circle, dotted line) respectively (r2 = 0.94; 0.99 and 0.98). Each point corresponds to a single gel; lines are regression lines. (TIF) [file pone.0093325.s001.tif]

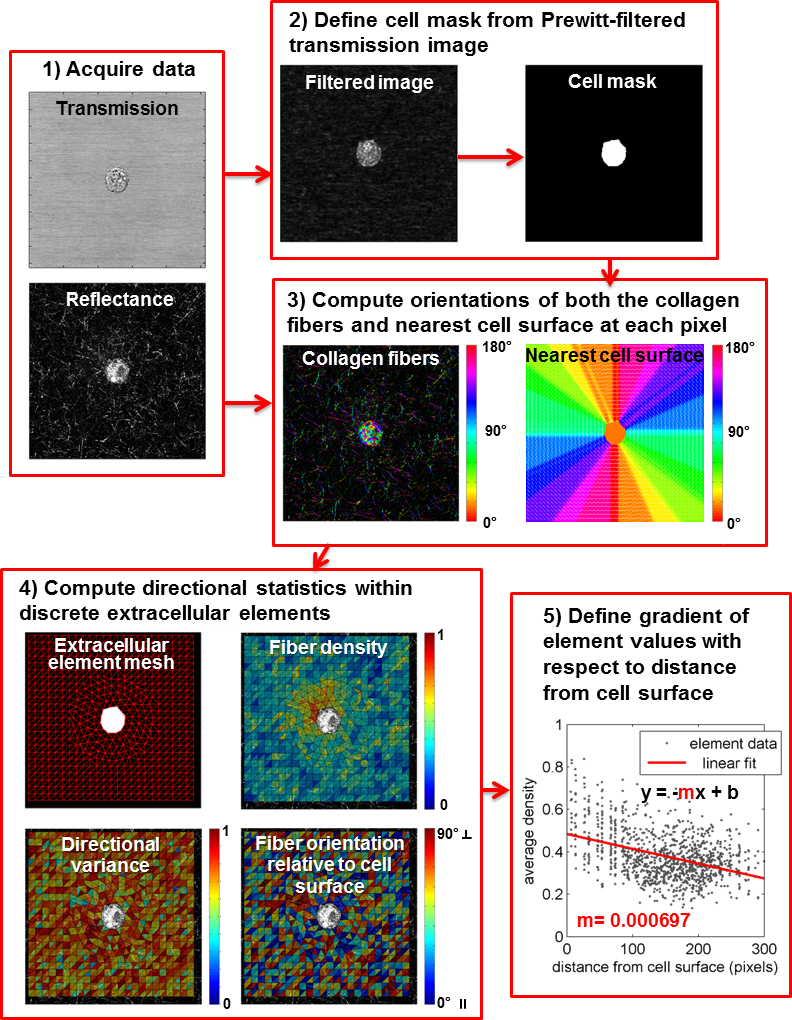

Supplement: Figure S2 — Overview of the image processing steps for quantifying fiber organization. Transmission and confocal reflectance images were used to isolate cell and fiber structures, respectively. Transmission images were filtered and segmented to define a cell mask, and the orientation relative to the nearest mask surface was defined for each pixel location. Fiber orientation was defined at each pixel as previously described [39], and directional statistics were computed within discrete extracellular regions defined by a triangular element mesh. The gradient of fiber data values with respect to the distance from the cell surface were computed using all extracellular elements, and the average fiber statistics from elements within just the first 40 μm from the cell surface were also computed. (TIF) [file pone.0093325.s002.tif]

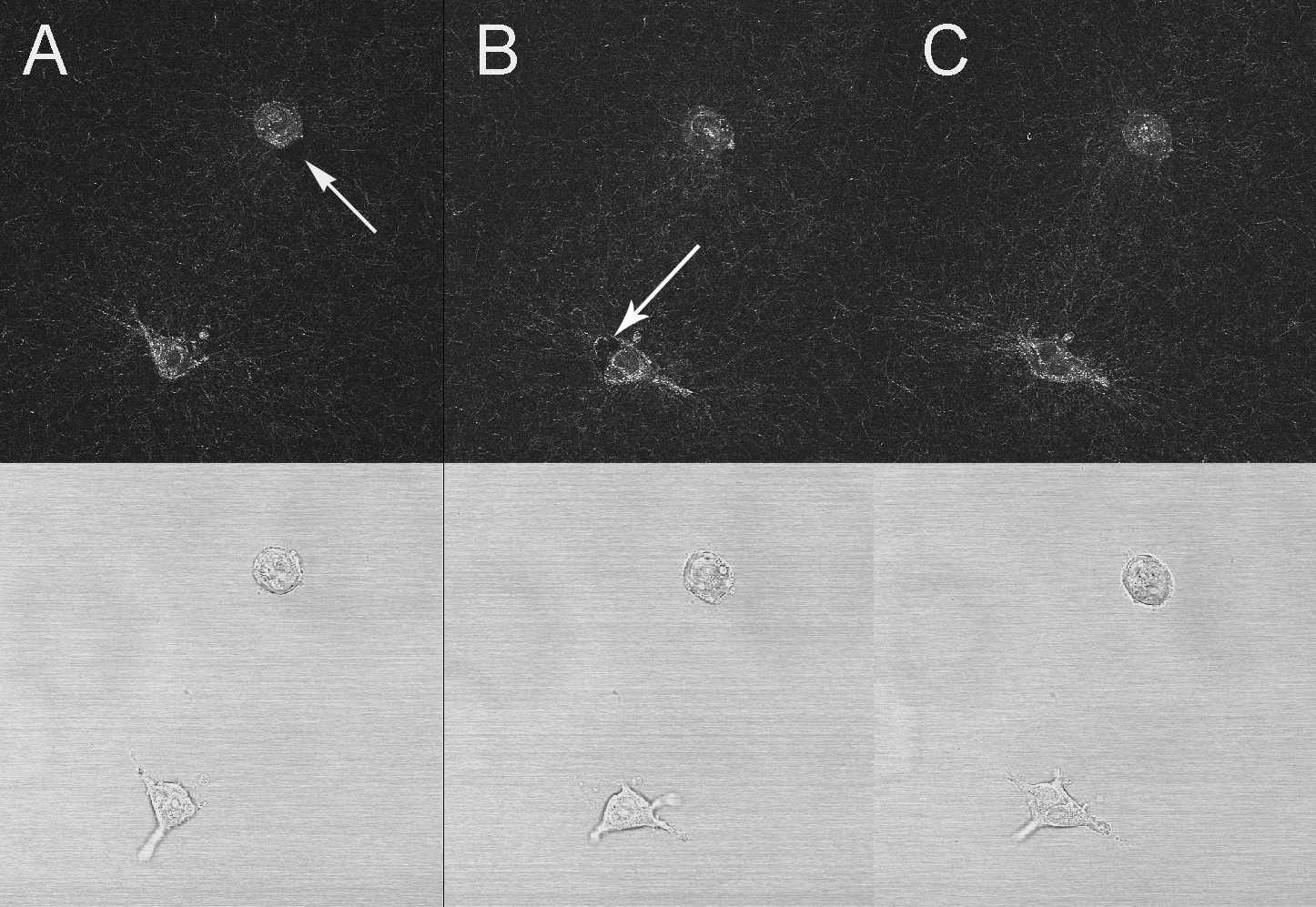

Supplement: Figure S3 — Collagen degradation during early stage morphogenesis. Reflection confocal (top row) and brightfield (bottom row) images taken at 8, 9 and 10 hours post-seeding. (A) At 8 hours the cell at the center of the field has degraded an area of collagen (arrow) while the lower cell has a cell protrusion extending towards the upper left. (B) At 9 hours the cell at the center begins to move into the degraded collagen area while the lower cell moves to produce a protrusion pointing to the lower right revealing an area of degradation (arrow). (C) At 10 hours the cell at the center, as well as the one below, now inhabit zones of degradation previously revealed, indicating continuous movement into and out of these zones. (TIF) [file pone.0093325.s003.tif]
